# Toxicity of a dental adhesive compared with ionizing radiation and zoledronic acid

**DOI:** 10.4317/medoral.20259

**Published:** 2015-06-02

**Authors:** Miguel Alcaraz, Amparo Olivares, Daniel-Giyngiri Achel, Emilio García-Cruz, Adriana Fondevilla-Soler, Manuel Canteras-Jordana

**Affiliations:** 1 M.D.,Ph.D., Senior Lecturer, Radiology and Physical Medicine Department, Faculty of Medicine/Dentistry, University of Murcia, Murcia, Spain; 2 D.D.S.,Ph.D., Assistant Professor, Radiology and Physical Medicine Department, Faculty of Medicine/Dentistry, University of Murcia, Murcia, Spain; 3 M.D.,Ph.D., Contracted Doctor, Applied Radiation Biology Centre, Radiological and Medical Sciences Research Institute, Ghana Atomic Energy Commission, Legon-Accra, Ghana; 4 M.D., Clinical Associate Professor, Stomatology Department, Faculty of Medicine/Dentistry, University of Murcia, Murcia, Spain; 5 M.D.,Ph.D., Radiotherapy Service, Unit of Head and Neck Cancer, Radiotherapy Service, Onclogy Institute of Madrid, Murcia, Spain; 6 M.D.,Ph.D., Senior Lecturer, Biostatistics Department, Faculty of Medicine/Dentistry, University of Murcia, Murcia, Spain

## Abstract

**Background:**

To determine the toxicity of aqueous dilutions of a universal self-priming dental adhesive (DA) and comparing these with those elicited by exposure to ionizing radiation (IR), Zoledronic acid (Z) treatment and the synergic effects of the combined treatment with IR+Z.

**Material and Methods:**

The genotoxic effect of DA was determined by the increase in the frequency of micronuclei in cytokinesis-blocked in cultured human lymphocytes before and after exposure to 2Gy of X-rays. The cytotoxic effect was studied by using the MTT cell viability test in normal prostate cell lines (PNT2) after exposure to different X-ray doses (0Gy-20Gy). The cell lines divided into different groups and treated with different test substances: DA in presence of O2, DA in absence of O2, Z-treated and control.

**Results:**

An in vitro dose-dependent and time-dependent cytotoxic effect of DA, Z and IR on PNT2 cells (*p*>0.001) was demonstrated. DA without-O2, following the recommendations of manufacturers, had a more pronounced effect of increasing cell death than DA with-O2 (*p*<0.001). In the genotoxicity assay, DA at 25% of its original concentration significantly increased chromosome damage (*p*<0.001). The samples studied were found to be toxic, and the samples photo-polymerized in absence of O2 showed a bigger cytotoxic effect comparable to the additive toxic effect showed by the combined treatment of IR+Z.

**Conclusions:**

Additional effort should be carried out to develop adhesives, which would reduce the release of hazardous substances; since toxic effects are similar to that reported by other agents whose clinical use is controlled by the health authorities.

**Key words:** Micronucleus, toxicity, dental adhesive, zolendronic acid, radiation effects.

## Introduction

Resin-based composite dental materials are widely used in dentistry. Their toxicological effects reported thus far have been attributed to the release or residual monomers or other substances derived either from incomplete polymerization or resin degradation. More than 30 different compounds have been identified as eluates from polymerized dental composites including major resin monomers, compomers, additives and degradation products, each with potentially different cytotoxic capabilities (1).

However, there is quite a limited knowledge concerning the genotoxic and mutagenic effects of any of the components released or eluted from widely used commercially available dental composite resin and adhesive systems. Monitoring the genotoxicity and cytotoxicity of these eluates would provide a better understanding of their interaction with the oral tissues and secretions as well as offer an *in vivo* approach to evaluate their potential toxicological effects (genotoxicity and cytotoxicity) (2).

Even though recommendations made by the manufacturer shows that the indirect laboratory-processed composite resin systems would be less cytotoxic and genotoxic when extensive polymerization occurred in the absence of oxygen during the setting reaction, some components of restorative composite resins are believed to be released in the oral environment initially during polymerization reaction and later due to degradation of the material. Some authors have proposed that cell culture toxicity data are highly model dependent and that internationally standardized test protocols for toxicity screening of dental materials in line with the existing standards are clearly needed to obtain comparable results (3).

Really, we found no previous studies comparing the toxicity of these dental materials with other physical and chemical agents that are clearly established as mutagenic and cytotoxic agents and which would allow the toxicity of these dental adhesives to be brought into perspective. In this study, we compared the cytotoxic and genotoxic effects induced by one of the most widely used adhesives in dental clinics today with those induced by two other known toxic and mutagenic agents: exposure to ionizing radiation and treatment with therapeutic doses of Z. For this, we performed a thorough polymerization of an adhesive resin in saline, evaluating its cytotoxic and genotoxic effects while it was under store in the in saline environment for months.

## Material and Methods 

- Chemicals and Reagents.

Zoledronic acid (Z) (Zometa®) was obtained from Novartis Pharmaceuticals (Barcelona, Spain). Universal self-priming dental adhesive (DA) (Prime&Bond® NT) was obtained from DENTSPLY©. RPMI 1640, F10, PHA, DMSO, cytochalasin B, streptomycin, penicillin, phosphate buffered saline (PBS) and 3-(4,5-dimethyl-2-thiazolyl)-2,5-diphenyl-2h-tetrazolium bromide (MTT), were obtained from Sigma-Aldrich Chemicals S.A (Madrid, Spain). Fetal bovine serum was obtained from Gibco (USA); glacial acetic acid and ethanol were obtained from Scharlab SL (Madrid, Spain), methanol was obtained from Pancreac (Madrid, Spain); 5% sodium heparin was obtained from Rovi Laboratories (Madrid, Spain) and 95 % Rosmarinic acid (RA) was obtained from Extrasynthese (Genay, France).

Z was dissolved in physiological saline (B. Braun Medical, S.A., Madrid, Spain) following the manufacturer´s instructions for therapeutic use to 5% (Z5). Two drops of self-priming DA adhesive were dispensed into the well. It was polymerized with the aid of halogen light Optilux 501® (SDS Ker, Scafatti, Italy), at an intensity of 350 mW/cm2 for 20 seconds at times in the presence of oxygen (With-O2) and in others, depriving the samples of oxygen (Without-O2), thus obtaining a total of 3,907 grams of polymerized product which was immersed in 100 ml of saline. The sample was stored at room temperature and 10 ml of saline were withdrawn at different times (1 hour, 24 hours, 1 day, 3 weeks) which were frozen at -80 °C until use. RA was also dissolved in physiological saline and mixed with blood to obtain a final concentration of 25 µM.

- Cell survival curve and viability quantification, MTT test.

The MTT assay has been extensively used to assess the cytotoxicity of dental materials and indicates cell viability based on mitochondrial dehydrogenase activity. It determines the degree to which cell proliferation is inhibited (4) and, in radiobiology, the cell survival rate against toxic agents (5,6).

- Cell line and culture conditions

The PNT2 cell line used was obtained from the European Collection of Cell Cultures (ECACC), Health Protection Agency Culture Collection (Catalogue nº 95012613, HPACC, UK). Tests were carried out to confirm the absence of *Mycoplama spp*. throughout the study. The PNT2 cells were cultured in RPMI 1640 supplemented with fetal bovine serum (FBS) (10%), glutamine (2 mM) and streptomycin plus penicillin (100 µg/ml and 100 IU/ml, respectively). All the cell culture processes were carried out in a Cultair ASB type II vertical laminar flow chamber. The PNT2 cultures were kept at 37ºC and 95% relative humidity, 5% CO2 atmosphere, in a Cytoperm incubator. The culture medium was changed every 2 days or when acidification was indicated by the pH indicator (phenol red). After irradiation or treatment, all micro plates were incubated for an additional 24h or 48h, and no medium changes were performed. DA assayed (With- and Without-O2) was administered at different volumes 25µl and 50µl; Z was administered at concentrations of 5 % and 100% (25µl).

- MTT test

To analyze for the effects of DA (With- and Without-O2), Z and IR on PNT2 cell viability and survival, we used the 3-(4,5- dimethylthiazol-2-yl)-2,5-diphenyl tetrazolium bromide (MTT) assay for 24 or 48 hours. Briefly, the cultures were incubated in 200 µl growth medium and allowed to adhere for 24 hours. After treatment at the above mentioned incubation doses of DA, Z and IR, and for the stated durations, supplemented growth medium and 50 µl of MMT (5 mg/ml) were added to each well in the 96 well plates and the micro plates were further incubated in a 5% CO2 atmosphere at 37°C for 4 hours. After centrifugation to carefully remove the medium and non-metabolized MTT, 100 µl of DMSO was added to each well to solubilize the MTT produced by the cultured cells. After shaking for 30 min at room temperature, the plates were read with a Multiskan MCC/340P spectrophotometer using 570 nm for the test reading and 690 nm as the reference wavelength. The negative control well was used for the baseline zero. Each experiment was repeated on three occasions and the results of all micro plates were obtained via automated and independent analysis.

- Genototoxic Effect: MNCB assay

The cytokinesis-block micronucleus assay (CBMN) is the most commonly used assay for determining chromosomal damage and the mutagenic capacity of chemical or physical genotoxic agents (7-12).

- Blood samples and irradiation procedure

Human peripheral blood samples were obtained from six healthy young nonsmoking female donors into heparinized tubes. For the non-irradiation treatments 20 µl of 25% DA solution, 20 µl of 5% Z and 100% of Z solutions were added to 2 ml of human blood. For the X-irradiation treatments 20 µl of these solutions were added to 2 ml of human blood and the samples were homogenized just before X-irradiation.

The study and informed consent documents were approved by local ethics committee in Biomedical Research (Committee of Ethics in Research at the University of Murcia, Spain). All participants gave written informed consent for the use of their blood samples in the study.

- Culture technique

The micronucleus (MN) assay was carried out on the cultured human lymphocytes, with the cytokinesis-block micronucleus method (MNCB) as described by Fenech and Morley (1985) (8). Briefly: a whole blood samples (0.5 ml) was cultured at 37°C for 72 hours in 4.5 ml of F-10 medium containing 15% fetal bovine serum, 1.6% (µg/ml) phytohaemaglutinin, 1% penicillin/streptomycin and 1 µg/ml of glutamine. Forty-four hours after initiation of the lymphocyte culture, 150 µl of cytochalasin B was added at a concentration of 3 µg/ml (6 µg/ml). After 72 hours the lymphocytes were treated with hypotonic solution (0.075 M KCl) for 3 min and fixed using methanol: acetic acid (3:1). Air-dried preparations were made and slides were stained with May-Grünwald Giemsa 24 hours later. Each experiment was repeated on three occasions.

- Scoring of Micronucleus

Triplicate cultures were analysed for each substance. In each, at least 3000 cytokinesis-blocked cells (CB cells) (MN/500 CB) were determined by two specialists who analyzed the slides of all groups via a double-blind method using a Zeiss light microscope (Oberkochem, Germany) with 400 X magnification to examine the slides and 1000 X magnification to confirm the presence or absence of MN in the cells (3000 CB/sample studied), according to the published criteria (7,8).

- Irradiation

The samples were exposed to X-rays with an Andrex SMART 200E instrument (YXLON International, Hamburg, Germany) operating at 200 kV, 4.5 mA, 36 cm FOD, at room temperature. The radiation doses were monitored by a UNIDOS® Universal Dosimeter with PTW Farme® ionization chambers TW 30010 (PTW-Freiburg, Freiburg, Germany) in the radiation cabin and the dose of radiation of X-rays was confirmed by means of thermoluminescent dosimeters (TLDs) (GR-200-, Conqueror Electronics Technology Co. Ltd, China). The CIEMAT (Ministry of Industry and Energy, Spain) supplied the TLDs and also measured their absorbed doses after the experiments. In the cytokinesis-blocked micronucleus test (MNCB) using human lymphocyte cells, 2 Gy of irradiation was administered, whereas different doses of X-rays (4Gy, 8Gy, 12Gy, 16Gy, 20 Gy and 0Gy as control) were used in the PNT2 cell viability assay.

- Statistical analysis

In the genotoxicity study, the degree of dependence and correlation between variables was assessed using analysis of variance complemented by a contrast of means (p<0.05). Quantitative means were compared by regression and linear correlation analysis.

In the cytotoxicity assay, an analysis of variance (ANOVA) of repeated means was carried out to compare the percentages of surviving cells in the cultures with different concentrations of the various compounds, complemented by least significant deference analyses to contrast pairs and means. The analyses were carried out by logarithmically transforming the data to comply with ANOVA conditions. Groups of the same size were compared, a power of 80%, and a significance level of 5%.

## Results

- X-rays radioprotective effects: growth inhibition

**Figure 1:**
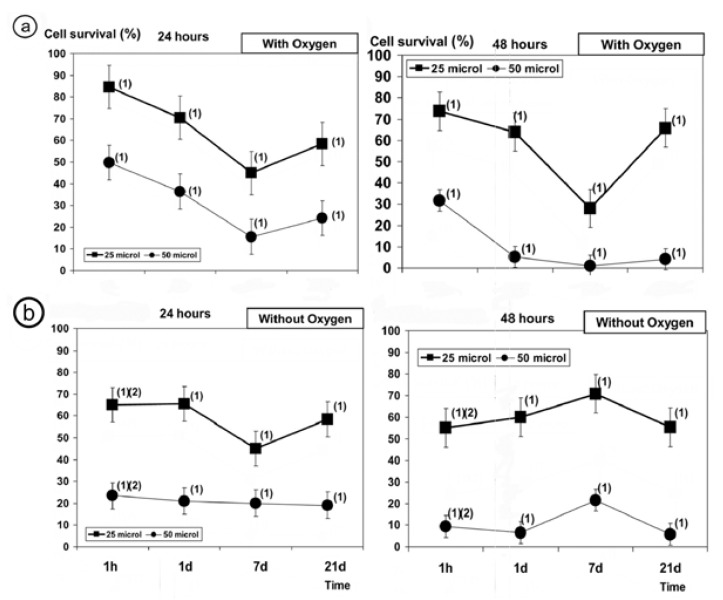
Cytotoxic effect of samples of Dental Adhesive on PNT2 cell viability after 24h and 48h of incubation : a) cell survival after administration of 25µl and 50µl of sample DA with-O2; b) cell survival after administration of 25µl and 50µl of sample DA without-O2 (1): (*p*<0.001) versus non irradiated control).

In the cytotoxicity study, the treatment of PNT2 cells with increasing volumes (25µl and 50µl) of DA (with-O2 and without-O2) for 24 and 48 hours caused a dose-dependent and time-dependent decrease in cell survival (*p*<0.001) and showed a significant degree of cytotoxicity (Fig. 1). Using the manufacturer´s recommendation, i.e., administration of DA (without-O2) (Fig. 1b) determined 1h, for 24 and 48 h and at both volumes (25µl and 50 µl), cause an increment in the reduction in cell survival (*p*>0.001), greater than the treatment of DA with-O2 was noticed (Fig. 1a).

**Figure 2:**
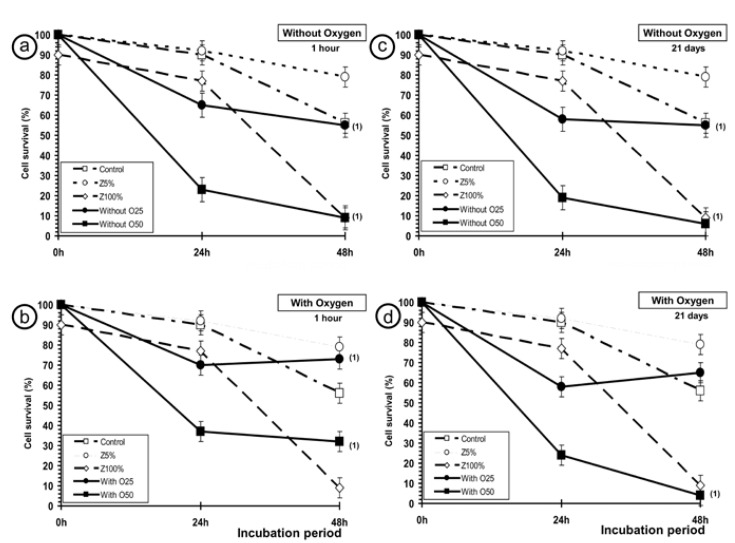
Cytotoxic effect of samples of Dental Adhesive on PNT2 cell viability after 24h and 48h of incubation compared with the cell survival of irradiated controls, treated with 25µl of zoledronic acid at 5% concentrations (Z5%) and treated with 25µl of zoledronic acid at 100% concentration (Z100%) at the beginning (1 hour) and end of the study (21 days) period: a) after 1 hour of DA without-O2 (25µl and 50µl); b) after 1 hour of DA witht-O2 (25µl and 50µl); c) after 21 days of DA without-O2 (25µl and 50µl); d) after 21 days of DA witht-O2 (25µl and 50µl) ( (1): (*p*<0.001) versus irradiated controls).

Figure 2 shows the synergic effect on the decrease in cell survival when different concentrations of Z were combined with IR and a cytotoxic effect of DA with-O2 and DA without-O2. The cytotoxic effect determined 1h after photo-polymerization is similar to the decreased cell survival noticed in the treatment with IR: 25µl of DA whitout-O2 was similar to 20Gy of X-rays and 50µl of DA without-O2 was similar to combined treatment of Z100% + 20Gy of X-rays (Fig. 2a). It can also be seen that the cytotoxic effect after 1 hour (Fig. 2a) of photo-polymerization is only slightly higher than the cytotoxic effect obtained after 21 days (Fig. 2c). Finally, the samples of DA with-O2 determined 1h after photo-polymerization (Fig. 2b) were less cytotoxic than those obtained with DA without-O2 which was considered as following the manufacturer’s recommendations. However, the samples of DA with-O2 (50 µl) determined 21h after photo-polymerization was similar to the decreased cell survival to the samples whitout-O2, similar to combined treatment of Z100% + 20Gy of X-rays (Fig. 2d).

- X-Ray genoprotective effects: Antimutagenic activity.

In the genotoxic study, the basal micronuclei frequency was 10±2 MN/500 CB for the non-irradiated control blood samples. Irradiation with 2 Gy produced a significant increase in the appearance of MN, which reached 26±4 MN/500 CB (*p*<0.001), and expresses a genotoxic damage induced by the X-rays. The administration of RA, used as positive control of a radio protective agent, led to a significant drop in the frequency of MN when administered before (*p*<0.001) X-irradiation, which expresses a genoprotective capacity against chromosome damage induced by X-ray.

**Figure 3:**
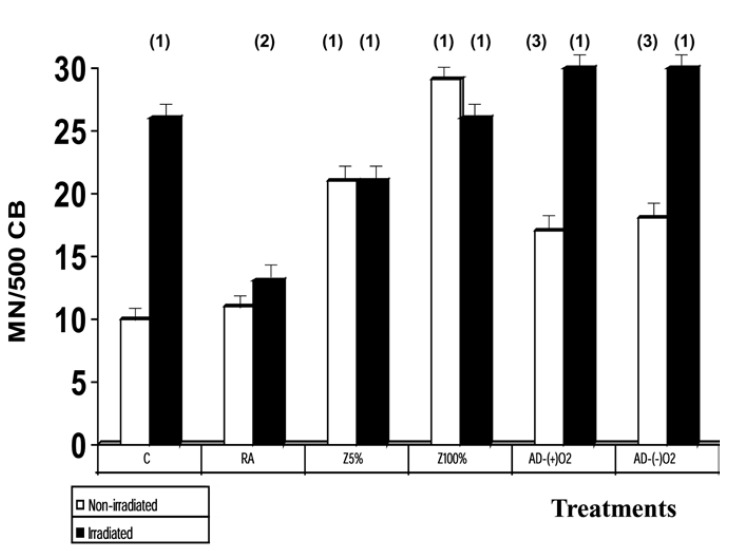
Genotoxic effect (frequency of MN/500CB) of samples of dental adhesive administered before and after the irradiation with 2 Gy of X-rays (C, control; RA, rosmarinic acid; Z5%, zoledronic acid at 5% concentration; Z100%, zoledronic acid at 100% concentration; DA-O2, sample of dental adhesive in presence of oxygen; DA-O2, sample of dental adhesive in absence of oxygen (1): (*p*<0.001 versus non irradiated control; (2): (*p*<0.001) versus irradiated control; (3) (*p*<0.01) versus non irradiated control).

The administration of the samples of DA (with-O2 and without-O2) caused the death of the lymphocytes making it impossible to conduct the micronucleus assay. The concentration that allowed appropriate micronucleus test was 25% of the original concentration. Under these conditions, the administration of DA with-O2 and DA without-O2 induced a significant increase in the frequency of MN/500 CB (*p*<0.001) compared with the controls, which represents a genotoxic effect induced by DA (Fig. 3).

The administration of Z caused a dose-dependent increase in the frequency of MN/500 CB (p<0.001) compared with the controls, which represents a genotoxic effect induced by Z (*p*<0.001) (Fig. 3).

## Discussion

The MTT assay was employed for the assessment of the toxic effects of the agents/compounds on the cells. The MTT assay has been extensively used to asses cytotoxicity of dental materials and indicates cell viability based on mitochondrial dehydrogenase activities. In this test, methylthiazol tetrazolium is metabolically reduced to colored formazan. The factors that inhibit dehydrogenase activity affect this colour associated reaction. It has been shown that activated cells produce more formazan than resting cells; therefore, it is possible to measure cell activity or enzyme activities (4). The cytokinesis-block micronucleus assay (MNCB) on human lymphocytes which is based on the increase in the frequency of appearance of MN is the most commonly used assay for determining the mutagenic capacity of a chemical or physical genotoxic agents (5,7,9). Both assays have been used extensively to evaluate the genotoxic and cytotoxic effects of chemical substances and physical agents in toxicological tests.

Our results show significant dose-dependent genotoxic and cytotoxic abilities of the dilutions obtained from dental adhesive solutions tested for all the periods studied. Surprisingly, the polymerization of the adhesive scrupulously following the manufacturer’s instructions (described as DA without-O2) showed an increased cytotoxic capability although it would be considered that going by these directives, the photo-polymerization of the adhesive should be more complete.

Numerous studies have described the genotoxic potential of different Das (13) and their cytotoxic capacities on different cell lines. It has been described as follows cytotoxicity way: a) the short term, the release of free monomers that occur during the monomer to polymer conversion, b) long-term, the release of leachable substances which are generated by degradation and erosion over time. Additionally, ion release and bacterial growth at the interface between the restorative material and dental tissues could also cause tissue response (14-23).

ROS (reactive oxygen species) generation induces incomplete polymerization of the monomers or the degradation of polymers over time, and has been described as one of the mechanisms leading to the harmful cytotoxic and genotoxic effects on cells. In consonance with others authors, our results show that the cytotoxicity of dentin adhesive systems is higher at 24-48 hours after placement (22). Furthermore, these materials undergo hydrolysis over time in aqueous medium impairing adhesion and causing the generation of new free radicals which results in toxicity (23).

Some authors have proposed that cell culture toxicity data are highly model dependent and that internationally standardized test protocols for toxicity screening of dental materials in line with the existing standards are clearly needed to obtain comparable results (3). In our opinion, the standardized test protocols for toxicity screening are available; however we found no previous studies comparing the toxicity of these dental materials with other physical and chemical agents that are clearly established as mutagenic and cytotoxic agents, which would allow the toxicity of these dental adhesives to be brought into real perspective. This allows us to study its toxic effects on the human organism once the toxic substances produced extend to the rest of the organism via the circulatory system, either via the pulp or intestinal or digestive absorption.

The genotoxicity test has been described in cultured human lymphocytes exposed to radiation and is considered the ideal assay for measuring chromosomal damage and quantify the genotoxic capacity of such substances (5,7,9).

In the present study, human prostate epithelial cells were utilized due to their consideration as sensitive to radiotherapy techniques; also due to the use of zoledronic acid in bone metastases in human prostate tumours, which in previous research allowed us to describe the different systemic responses to isolated or combined treatments using either one agent alone or both jointly (Z + IR). As such, the results we have determined regarding the toxicity of dental adhesives (DA) would allow preliminary assessments to be made on their effects on the human organism as well as to establish a comparison on the known toxic effects induced by IR and Z.

Both assays have been used extensively to evaluate the genotoxic and cytotoxic effects of chemical substances and physical agents in toxicological tests.

In this regard, our study compares the effects induced by one of the most used adhesives in dental clinic today with those induced by two other known toxic and mutagenic agents: ionizing radiation exposure and treatment with therapeutic doses of Z.

Ionizing radiations (IR) causes the generation of a high level of hydroxyl radicals *in vivo*, through the hemolytic cleavage of body water or of endogenous hydrogen peroxide (formed by reduction of the superoxide anion) by two mechanisms: the Haber-Weiss and Fenton models. The hydroxyl radical is the most cytotoxic of all these so far described, with an estimated half-life of 10-9 s. the high reactivity of this radical implies immediate reaction at the place where it is generated (5,7). Thus, when hydroxyl radical generation is massive, as occurs during X-irradiation, the genotoxic and cytotoxic effect increases through the interaction of these radicals with cell phospholipid structures, inducing peroxidation processes and the generation of lipoperoxy radicals, which may be regarded as a delayed reaction by ionizing radiation (7,9). Currently, the ability of different substances to prevent genotoxic damage and their antimutagenic capacity is measured in terms of the production of these ROS (7,8). We have used the MN test to evaluate the genoprotective capacity of several compounds. In addition we described how some pure flavonoids (diosmin and apigenin) and polyphenolic extracts show a greater capacity than traditional radioprotectors, for example sulphur compounds (DMSO and AMF), against both X-rays in vivo (10,11) and γ-irradiation *in vitro* (7,12). We described how these protective capacities depend on the degree of polymerization and solubility of the substances assayed, since both modify their bioavailability (9-11). Reflecting the findings of other authors, we have observed that the antioxidant substances contained in different polyphenolic extracts of olive leaf (Olea europaea) (10,11) and citrus fruits (citroflavonoids) (10-12) show greater protective power when administered alone. Similarly, we used the MTT assay to study the cytotoxic capacity of ionizing radiation with different chemicals (6).

Zoledronic acid (Z), a third generation bisphosphonate, is used in the treatment of hypercalcaemia-related cancer (24), in complications for bone metastasis (25) and in post menopausal osteoporosis (25). It is more potent than nitrogenated bisphosphonates, acting on osteoblasts by inhibiting their chemotaxis, shortening their half life, slowing their activity and inducing apoptosis. As a consequence, it stops bone reabsorption (25). It also induces anti proliferative effects and apoptosis in different cell lines through the mitochondrial release of cytochrome C and activation of the caspase-3 pathway (26). Our study demonstrates the dose-dependent and time-dependent cytotoxic effect and genotoxic effect of Z on PNT2 cells *in vitro*. These results are consistent with earlier reports that Z can inhibit cell proliferation in different tumor cells (8,27,28). After correction for the drug’s toxicity using the therapeutic concentrations recommended for humans (5%), a synergic cytotoxic effect was identified with IR but with the characteristics of a powerful chemical radio sensitizing agent (26). Other authors have described a synergic effect with IR in several tumour lines (26-28) and described a sensitizing effect of Z, which could be used to reduce the doses used in oncological radiotherapy.

Our results obtained with aqueous samples in the presence of the dental adhesive show both genotoxic and cytotoxic capacities greater than the synergy of the two known mutagens (IR+Z), conducting our experiments with strict adherence to the manufacturer’s directives. Although, degradation of dental resins in human saliva, leading to a decrease in mechanical strength and surface hardness, have been reported in different in *in vitro* studies (29), the results of these studies suggested that such degradation arose from the chemical degradation of methacrylate polymers due to enzyme-catalyzed hydrolysis of their ester bonds (30).

However, the genotoxic and cytotoxic capacities of dental adhesives is seems to be an acceptable side effect in the absence of other less toxic materials. According to Hagio *et al*., (2006) (30), the toxicities of hydrolyzed products are still unidentified and so they recommend further studies on the biocompatibility of hydrolyzed products.

We determined greater cytotoxic and genotoxic effects for DAs than those determined for a range of IR doses and of high doses of Z.Both agents (Z and RI) are subject to strict health and safety controls in terms of their use and handling due to their known toxicity. Surprisingly, the use of DA such as that analyzed in this study is freely available, under no form of regulation by the health authorities and their recommended forms of usage increment their toxicity.

In conclusion, the data presented in this paper and the results of other studies indicate that evaluated genotoxic and cytotoxic potential of dentin bonding agents might be a result of a toxicity of various components that are released from polymerized adhesives. As some genotoxic components of dentin bonding agents cannot be replaced by less dangerous ones, additional efforts should be made to develop adhesives with higher monomer polymer conversion, which would reduce the release of hazardous substances; since toxic effects are similar to that reported by other physical and chemical agent whose clinical use is controlled by the health authorities.
